# Radiation dose of prostatic artery embolization for benign prostatic hyperplasia: A protocol for systematic review

**DOI:** 10.1097/MD.0000000000031595

**Published:** 2022-11-18

**Authors:** Jinglei Liu, Kai Zhang, Xiaolong Wu, Bao Cui, Zhihui Liang

**Affiliations:** a Department of Interventional Treatment, 980 Hospital of PLA Joint Logistics Support Forces (Bethune International Peace), Shijiazhuang, China; b Department of Oncology, Shijiazhuang People’s Hospital, Shijiazhuang, China; c Department of Radiotherapy, First Affiliated Hospital of Zhengzhou University, Henan, China.

**Keywords:** benign prostatic hyperplasia, prostatic artery embolization, radiation, systematic review

## Abstract

Increasing evidence supports the efficacy and safety of prostatic artery embolization (PAE) in the treatment of benign prostatic hyperplasia. However, PAE relies on ionizing radiation, which has not been studied systematically so far. Therefore, the potential associated risks remain largely unknown and are subject to intense debate. We performed a protocol for systematic review and meta-analysis to evaluate the clinical benefits of different radiation doses in patients with benign prostatic hyperplasia undergoing PAE.

**Methods:**

A comprehensive search of several databases from 1966 to October 2022 was conducted. The databases include Ovid Medline In-Process & Other Non-Indexed Citations, Ovid MEDLINE, Ovid EMBASE, Ovid PsycINFO, Ovid Cochrane Central Register of Controlled Trials, Ovid Cochrane Database of Systematic Reviews, and PubMed. Risk of bias of the included studies was assessed by the “Risk of Bias Assessment Tool” of the Cochrane Handbook for randomized controlled trials. All data were analyzed using the Comprehensive Meta-Analysis software package (Biostat, Engelwood, NJ).

**Results:**

The results will be submitted to a peer-reviewed journal once completed.

**Conclusion:**

This review will provide reliable evidence for extensive application of PAE for benign prostatic hyperplasia and determine the most rational radiation dose for these patients.

## 1. Introduction

Benign prostatic hyperplasia is one of the most common diseases in men and is often associated with bladder outlet obstruction and lower urinary tract symptoms (LUTS), which can reduce quality of life by impeding normal activities and causing complications such as urinary retention, urinary tract infections, bladder stones, and renal insufficiency.[1–3] The incidence of benign prostatic hyperplasia in men aged 50 to 60 years is 50% and rises with increasing age.[4,5] Treatment options for patients with moderate to severe symptoms caused by benign prostatic hyperplasia are varied and include catheterization, medical therapies, minimally invasive therapies, and surgical therapies.[6,7]

Although transurethral resection of the prostate (TURP) is the most common surgical modality for benign prostatic hyperplasia and the reference standard, TURP is limited to prostates <80 mL and associated with a substantial complication rate.[8] Several therapeutic modalities that involve minimally invasive therapies including laser treatment, transurethral microwave thermotherapy, transurethral needle ablation, prostatic stent, intraprostatic injection of botulinum toxin or emergent materials, and prostatic artery embolization (PAE) have been introduced.[9]

PAE is a new endovascular technique that can serve as an alternative to more invasive procedures.[10] The effect of PAE is based on multiple impact mechanisms. Embolization causes displacement of intraprostatic vessels and precapillary arterioles, resulting in irreversible ischemia.[11] Increasing evidence supports the efficacy and safety of PAE in the treatment of lower urinary tract symptoms associated with benign prostatic hyperplasia.[12] However, PAE relies on ionizing radiation, which has not been studied systematically so far. Therefore, the potential associated risks remain largely unknown and are subject to intense debate. We performed a protocol for systematic review and meta-analysis to evaluate the clinical benefits of different radiation doses in patients with benign prostatic hyperplasia undergoing PAE, and use the findings to project, and articulate what might be considered as the optimal radiation dose for these patients.

## 2. Methods

### 2.1. Study registration

The protocol was designed in accordance with the Preferred Reporting Items for Systematic Reviews and Meta-Analysis guidelines extension for reporting systematic review protocols (PRISMA-P).[13] The review protocol was registered with the International Prospective Register of Systematic Reviews (PROSPERO), registration number (CRD42020159541). Ethical approval was not required for this study as all the research materials are derived from published studies.

### 2.2. Inclusion criteria

Studies with the following criteria are included:

(1) proven diagnosis of benign prostatic hyperplasia;
(2) patients require treatment due to moderate-to-severe LUTS;
(3) patients underwent PAE with different dose of radiation exposure;
(4) studies measured the pre- and post-PAE outcomes such as international prostate symptom score, quality of life score, international index of erectile function score, maximum urinary flow rate/peak urinary flow rate, postvoid residual volume, prostate volume, prostate specific antigen level; and
(5) randomized controlled trial.

The following studies were excluded:

(1) reviews, letter to editor, comments, studies published in languages other than English or Chinese;
(2) studies with less than 10 patients or animal studies;
(3) studies with insufficient data even after contacting the author, such as missing standard deviation, or the data shown in figures did not retrieve the exact number;
(4) studies showing duplicate data of the same patients; and
(5) studies including patients with suspicion of prostate cancer, hypocontractile bladder, or other neurogenic bladder disorders.

Studies which were published in multiple reports about the same sample were included once.

### 2.3. Search methods

**Figure 1.**
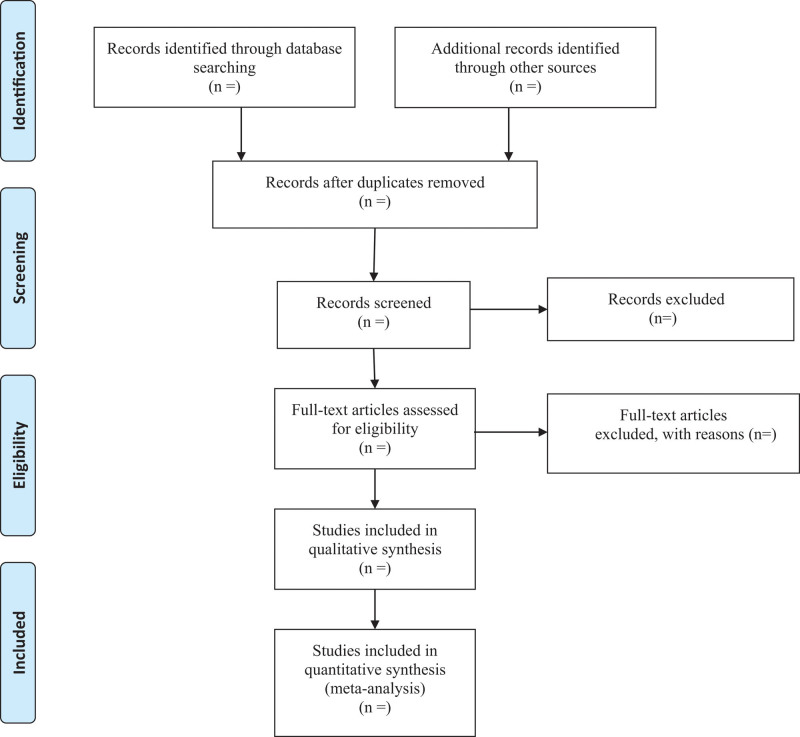
Flow diagram of study selection process.

A comprehensive search of several databases from 1966 to October 2022 was conducted. The databases include Ovid Medline In-Process & Other Non-Indexed Citations, Ovid MEDLINE, Ovid EMBASE, Ovid PsycINFO, Ovid Cochrane Central Register of Controlled Trials, Ovid Cochrane Database of Systematic Reviews, and PubMed. Search strategy for PubMed was shown in Table 1. Two authors will independently draft and carry out the search strategy. In addition, we manually retrieve other resources, including the reference lists of identified publications, conference articles, and gray literature. The key terms used for the search are “benign prostatic hyperplasia,” “lower urinary tract symptoms” and “prostatic arterial embolization.” The selection process of eligible papers is shown in a PRISMA flow diagram (Fig. 1).

**Table 1:** Search strategy for PubMed.

#1 Benign prostatic hyperplasia [MeSH Terms]
#2 Lower urinary tract symptoms [Title/Abstract]
#3 BPH [Title/Abstract]
#4 Prostatomegaly [Title/Abstract]
#5 Urinary obstruction[Title/Abstract]
#6 Bladder outlet obstruction [Title/Abstract]
#7 Urinary retention [Title/Abstract]
#8 LUTS [Title/Abstract]
#9 Benign prostate disease [Title/Abstract]
#10 #1 OR #2 OR #3 OR #4 OR #5 OR #6 OR #7 OR#8 OR#9
#11 Prostatic artery embolization [MeSH Terms]
#12 Minimally invasive surgery [Title/Abstract]
#13 Intervention surgery [Title/Abstract]
#14 Transcatheter arterial embolization [Title/Abstract]
#15 #11 OR #12 OR #13 OR #14
#16 #10 AND #15

LUTS = lower urinary tract symptoms.

### 2.4. Data extraction

Two review authors will independently extract the data and fill out the standard data extraction form, which includes study information such as the first author, publication year, title, journal name, research design, number of patients, inclusion criteria, interventions, control, treatment period, and outcome measures. Radiation dose is measured at the end of every PAE procedure and given as a dose area product. Data extraction will be performed by 2 independent investigators according to a predesigned review form. Disagreements are resolved through discussion among all authors.

### 2.5. Risk of bias assessment

Two reviewers will assess the risk of bias of the included studies by the “Risk of Bias Assessment Tool” of the Cochrane Handbook for randomized controlled trials.[14] The evaluation contents include random sequence generation, allocation concealment, blinding of participants and personnel, blinding of outcome assessment, incomplete outcome data, selective reporting, and other biases. Each item is divided into “high risk,” “unclear risk,” and “low risk.” Any inconsistencies will be determined in consultation with the third reviewer.

### 2.6. Statistical analysis

To perform the meta-analysis, all data were analyzed using the Comprehensive Meta-Analysis software package (Biostat, Engelwood, NJ). All outcomes were analyzed and presented with 95% confidence intervals in the form of mean difference using the random-effect model. Because eligible studies used various inclusion criteria and populations, the application of the random effect model is more suitable than the fixed-effect model. Sensitivity analysis was conducted to assess the heterogeneity of eligible studies and the impact of each study on the pooled effects. Additionally, heterogeneity between studies was checked by using the Q and I^2^ statistics and the *P*-value. To assess publication bias, a Begg funnel plot and Egger test were used. If significant publication bias was found, the fail-safe N and trim-fill tests were performed to confirm the degree of publication bias.[15] The results were considered statistically significant when *P* < .05.

### 2.7. Summary of evidence.

The assessment of evidence for all outcomes will be summarized using the Grading of Recommendations Assessment, Development and Evaluation (GRADE) approach.[16] The quality of evidence will be rated as high, moderate, low, and very low quality.

## 3. Discussion

Benign prostatic hyperplasia causing LUTS, such as a weak urinary stream, higher urinary frequency, intermittent voiding, nocturia, and urinary urgency, in elderly men is gaining more and more concerns nowadays and TURP still represents the gold standard of surgical treatment despite its considerable perioperative morbidity.[9,17] Recently, PAE was described as a novel effective and less invasive treatment alternative and evidence is growing including some prospective randomized controlled data which became available.[18] Whereas efficacy of PAE is accepted, controversial discussions centered on the radiation dose which PAE patients are exposed to and its inherent stochastic and deterministic effects during PAE treatment challenge us to seriously do a systematic review of the current available literature. This review study will provide reliable evidence for its extensive application and the most rational radiation dose for these patients.

## Author contributions

Bao Cui designed and reviewed protocol. Xiaolong Wu performed the data collection. Zhihui Liang performed the data analysis. Jinglei Liu and Kai Zhang finished the manuscript.

**Conceptualization:** Kai Zhang.

**Data curation**: Xiaolong Wu.

**Project administration**: Bao Cui.

**Writing – original draft**: Jinglei Liu.

**Writing – review & editing**: Zhihui Liang.

All of the authors approved the submission.
